# A SYSTEMATIC REVIEW OF CARE PARTNERS’ PERCEPTIONS OF FALL RISK AMONG OLDER PEOPLE WITH DEMENTIA

**DOI:** 10.1093/geroni/igad104.2560

**Published:** 2023-12-21

**Authors:** Nirali Thakkar, Yuanjin Zhou

**Affiliations:** University of Texas-Austin, Austin, Texas, United States; University of Texas at Austin, Austin, Texas, United States

## Abstract

Community-dwelling older people with dementia (cd-OPWD) experience a high risk of falling. Care partners have varied levels of involvement in fall risk management for cd-OPWD. However, little is known about what factors may shape their involvement. Understanding care partners’ perceptions of fall risk, associated factors, and impacts will be critical for service providers to effectively engage care partners in fall risk management for cd-OPWD. Utilizing the Common Sense Model of Illness Perceptions, we conducted a literature search among 6 research databases and identified 23 articles using predefined inclusion/exclusion criteria. Our study found that care partners’ perceptions of cd-OPWD’s fall risk comprised beliefs about fall risk management behaviors/programs (i.e., mobility aides, exercise, home modification, fall detector system), their understanding of indicators, causes, consequences, timeline, and controllability of cd-OPWD’s fall risk, and emotional reactions. These perceptions were influenced by care partners’ previous experiences with falls, cd-OPWD’s declining health and functions, experienced care burden, relationships with cd-OPWD, and interactions with healthcare providers. Care partners’ beliefs about fall risk management behaviors/programs and their understanding of indicators, causes, consequences, and timeline of falls are often associated with their adoption of fall risk management behaviors. Their emotional responses to cd-OPWD’s fall risk are associated with increased care burden. These findings point to a need for solution-oriented fall prevention interventions to increase care partners’ perceived benefits of fall risk management behaviors/programs and their knowledge about cd-OPWD’s falls. Future interventions should also try to mitigate the negative impact of care partners’ emotional responses to cd-OPWD’s fall risk.

